# Supplementary material for: Co-transfer of functionally interdependent genes contributes to genome mosaicism in lambdoid phages
Source: Microb Genom. 2022 Nov 24;8(11):mgen000915. doi: 10.1099/mgen.0.000915 (PMC9836094; doi:10.1099/mgen.0.000915)
Supplement: Supplementary material 1 [file mgen-8-915-s001.pdf]

Supplementary material

Supplementary figures

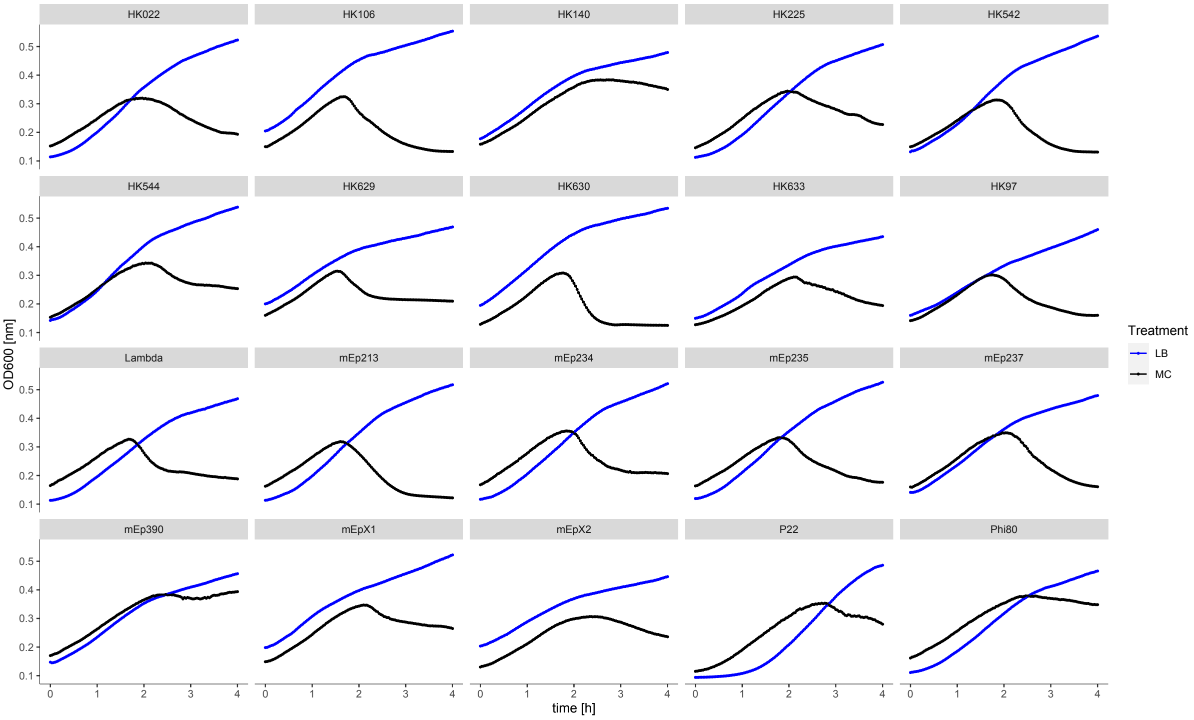

Figure S1: Induction curves for 20 of the focus phages. 4-hour growth curves in the absence (black) and presence of mitomycin C (black). The activity of the remaining 5 focus phages has been shown previously (see references in main text).

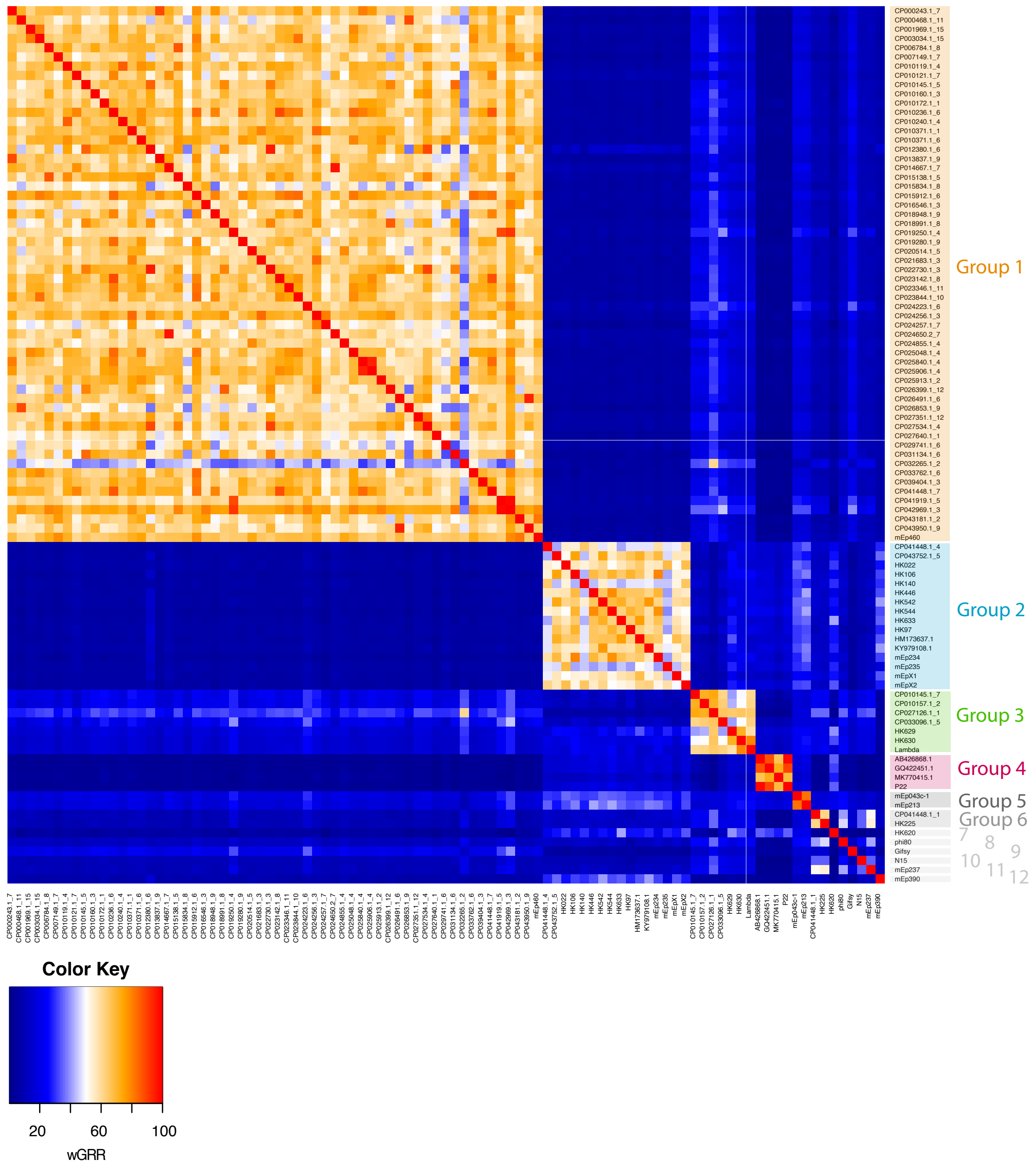

Figure S2: wGRR heatmap for 95 lambdoid phage genomes. The genome groups are highlighted by colors, where groups with less than 5 members are gray. Groups are sorted by size and the phages within each group are sorted alphabetically (case sensitive).

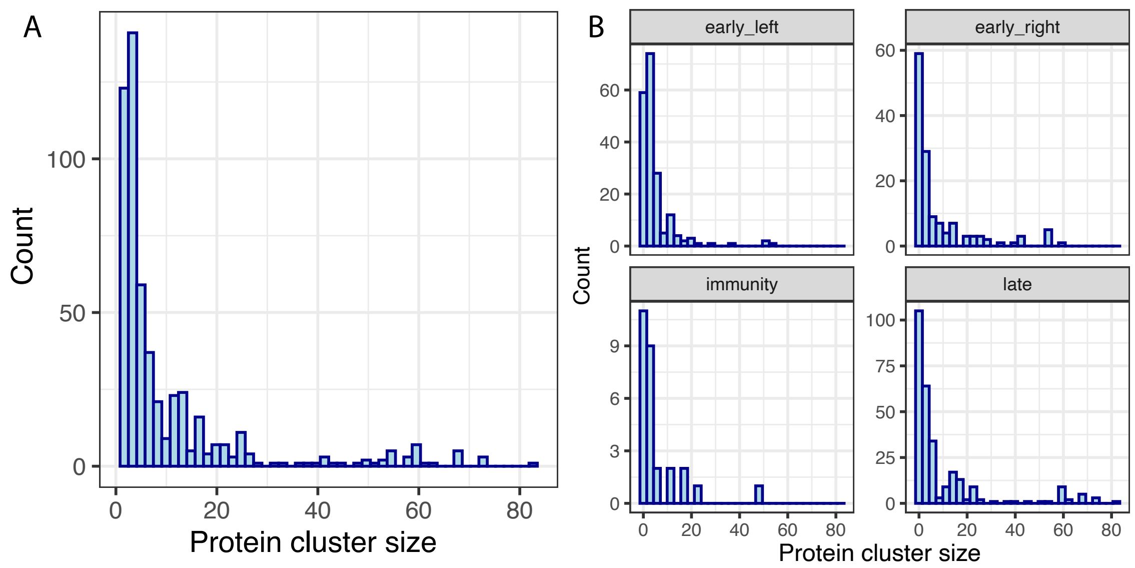

Figure S3: Protein cluster size distributions. A) Total protein cluster size distribution, singletons are excluded. B) Protein cluster size distribution per operon, singletons are included, proteins encoded in unknown operons are not shown. The protein cluster size distribution of each operon was compared to the protein cluster size distribution of the remaining operons using the Kolmogorov-Smirnov test. The early left operon tended to contain smaller protein clusters (D=0.1502, p=0.0045), the late operon tended to contain larger protein clusters (D=0.1089, p=0.0468), and the early right (D=0.0841, p=0.4307) and immunity (D=0.1680, p=0.4418) operons did not show a deviating size distribution.

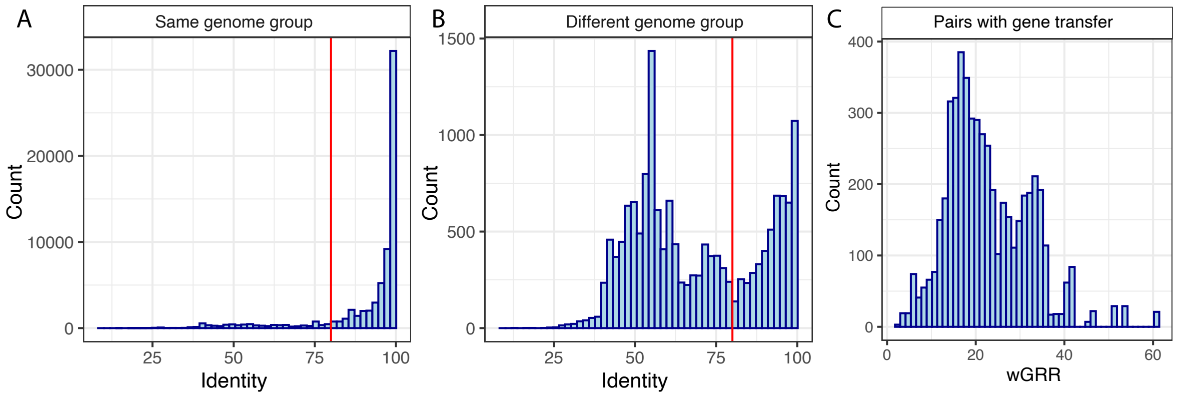

Figure S4: Cutoff for HGT detection. A,B) Histogram over pairwise protein identities within protein clusters from A) the same genome group or B) different genome groups. For the same genome group, we observe that 88% of the protein pairs in protein clusters have high identities (above 80%, red line). In contrast, only 5,238 (33%) of the protein pairs from different genome groups have identities above 80%. This motivates the identity cutoff of 80% that is used to detect transferred proteins. C) Histogram over wGRR for each transferred protein. Note that a genome pair can be included multiple times if multiple proteins have been transferred.

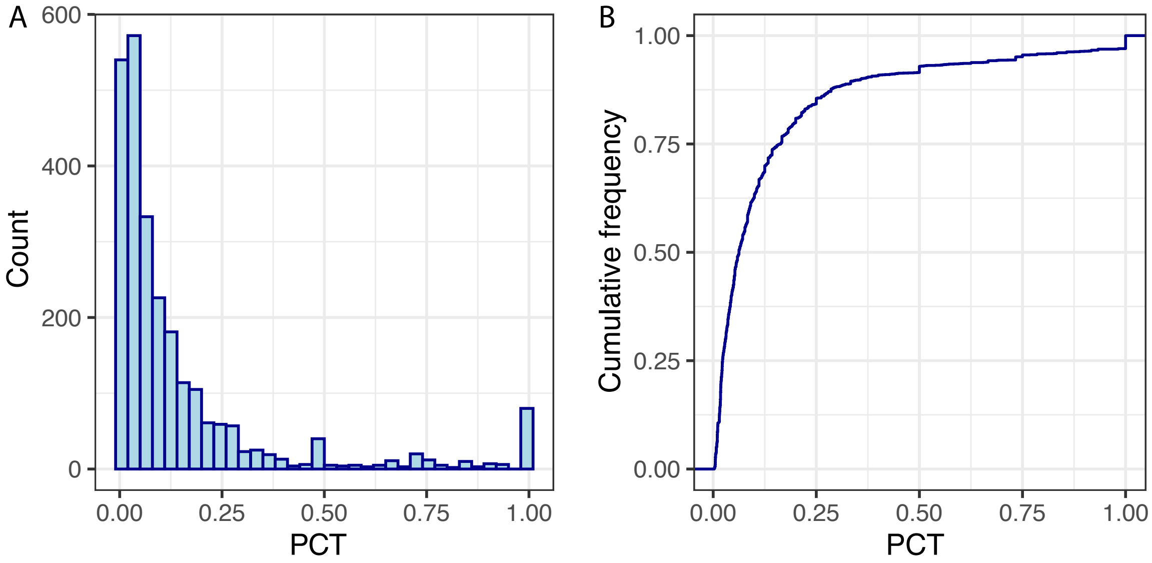

Figure S5: Distribution of protein cluster co-transfer (PCT) as A) histogram and B) empirical cumulative distribution function.

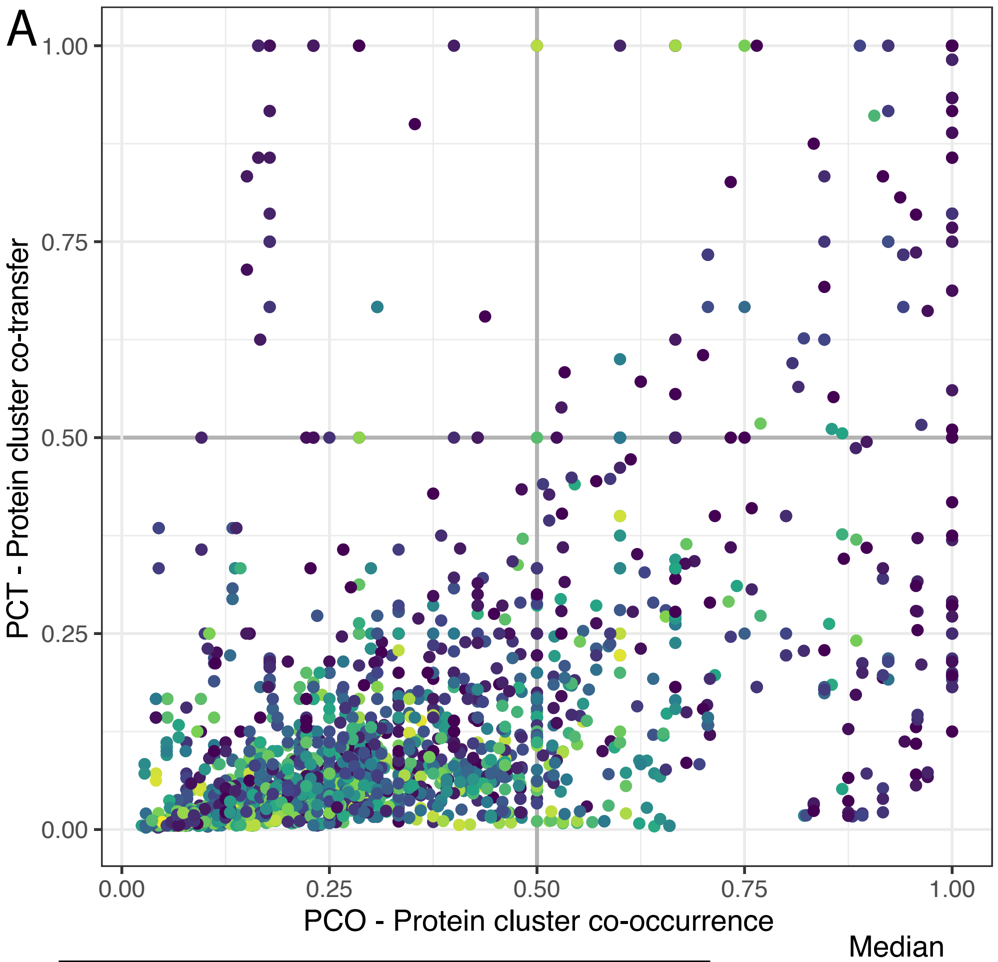

B

Category	Number	Median distance
PCT>0.5	181	2
PCO>0.5	500	5
PCT>0.5 & PCO>0.5	145	2
PCT>0.5 & PCO≤0.5	36	2
PCT≤0.5 & PCO>0.5	355	8

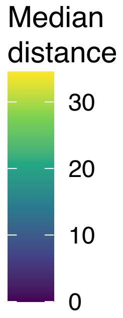

Figure S6: Protein cluster co-transfer (PCT) and protein cluster co-occurrence (PCO) for all protein clusters that are involved in co-transfer. A) Color-coded by median distance in number of genes, where the median is calculated across all genomes that contain both protein clusters. B) Number of frequent co-transfers (PCT>0.5), frequent co-occurrence (PCO>0.5) and combinations of these conditions. In addition, the median over all median distances in the category is listed.

Supplementary tables (in separate xlsx)

Table S1: Phage genomes included in the analysis. A) Focus phages. B) NCBI phages. C) *E. coli* prophages

Table S2: Genome annotation

Table S3: Pairs of transferred genes

Table S4: Number of transferred protein clusters per operon

Table S5: Pairs of protein clusters that are frequently co-transferred

Table S6: Modules of protein clusters involved in frequent co-transfers

Table S7: Pairwise similarities for Cro, CI, and Kil protein clusters. A) Within-cluster similarities. The proteins marked by a star (*) are involved in recombination. B) Between-cluster similarities. Only the pairs with a significant blast hit are considered for the average identity calculation.
